# Integrity of SRP RNA is ensured by La and the nuclear RNA quality control machinery

**DOI:** 10.1093/nar/gku761

**Published:** 2014-08-26

**Authors:** Eileen Leung, Claudia Schneider, Fu Yan, Hatem Mohi-El-Din, Grzegorz Kudla, Alex Tuck, Wiebke Wlotzka, Victoria A. Doronina, Ralph Bartley, Nicholas J. Watkins, David Tollervey, Jeremy D. Brown

**Affiliations:** 1 RNA Biology Group and Institute for Cell and Molecular Biosciences, The Medical School, Newcastle University, Newcastle upon Tyne NE2 4HH, UK; 2 Wellcome Trust Centre for Cell Biology, University of Edinburgh, Edinburgh EH9 3JR, UK

## Abstract

The RNA component of signal recognition particle (SRP) is transcribed by RNA polymerase III, and most steps in SRP biogenesis occur in the nucleolus. Here, we examine processing and quality control of the yeast SRP RNA (scR1). In common with other pol III transcripts, scR1 terminates in a U-tract, and mature scR1 retains a U_4–5_ sequence at its 3′ end. In cells lacking the exonuclease Rex1, scR1 terminates in a longer U_5–6_ tail that presumably represents the primary transcript. The 3′ U-tract of scR1 is protected from aberrant processing by the La homologue, Lhp1 and overexpressed Lhp1 apparently competes with both the RNA surveillance system and SRP assembly factors. Unexpectedly, the TRAMP and exosome nuclear RNA surveillance complexes are also implicated in protecting the 3′ end of scR1, which accumulates in the nucleolus of cells lacking the activities of these complexes. Misassembled scR1 has a primary degradation pathway in which Rrp6 acts early, followed by TRAMP-stimulated exonuclease degradation by the exosome. We conclude that the RNA surveillance machinery has key roles in both SRP biogenesis and quality control of the RNA, potentially facilitating the decision between these alternative fates.

## INTRODUCTION

Stable, non-coding RNAs are required for many key cellular processes, and largely function as components of ribonucleoproteins (RNPs). RNP assembly is facilitated, and coupled to maturation of the RNA component from precursor to mature form. In *Saccharomyces cerevisiae* (yeast) characterized nucleases, including the 5′-exonuclease Rat1, the 3′-exonucleases Rex1–3 and the exosome subunits Rrp6 and Rrp44, mediate many of these processing events (1,2). Rrp6 is a nuclear-restricted 3′-exonuclease, which also functions independently of the exosome. In contrast, Rrp44 has both 3′-exonuclease and endonuclease activities, is present throughout the cytoplasm and nucleus as part of the exosome complex and also functions in mitochondria (3–11). The activities of the nuclear exosome and Rrp6 are stimulated by complexes containing either the Trf4 (Pap2) or Trf5 poly(A) polymerases (TRAMP4 and TRAMP5 complexes), one of the two zinc knuckle proteins Air1 and Air2 and the helicase Mtr4 (12–15). Mtr4 is also required for several TRAMP-independent activities of the nuclear exosome, including 5.8S rRNA processing and degradation of the 5′ external transcribed spacer of pre-rRNA (16–18). The TRAMP and exosome complexes function both in regulated RNA processing during RNP biogenesis, and in RNA surveillance. It remains unclear how the exosome and its cofactors distinguish between RNAs that should undergo precisely regulated processing or be completely degraded.

Further factors important for maturation of many non-coding RNAs are the La protein (Lhp1 in yeast) and the nuclear Lsm2–8 complex. La is particularly important for correct processing of newly synthesized RNA polymerase III (pol III) transcripts, which it binds through their 3′-oligo(U) tracts (19–22). Yeast strains lacking Lhp1 are viable, but show defects in 3′ processing of pol III transcripts and other stable non-coding RNAs (23–25). La can stabilize newly synthesized transcripts, enabling maturation of defective tRNAs and U6 snRNA mutants that would otherwise be degraded, and has RNA strand annealing activity, indicating that it can act as an RNA chaperone (23,26–31). La is largely nuclear, and may retain RNAs in the nucleus (32–34). Indeed, deletion of a nuclear retention motif from La results in aberrant trafficking of the protein and concurrent defects in tRNA processing (35). Lsm complexes bind stably to several RNAs including the U6 snRNA and snR5 small nucleolar RNA (36,37). Like La, Lsm proteins are required for accurate processing of many non- RNAs including pol III transcribed tRNAs (26,38–40). Deletion of *LHP1* is synthetic lethal in combination with *LSM* gene deletions and with the *lsm8–1* conditional mutation, indicating that Lhp1 and the Lsm complex have overlapping functions (26,40).

The signal recognition particle (SRP) functions in co-translational targeting of presecretory and membrane proteins to the endoplasmic reticulum membrane (41,42). SRP comprises the pol III transcribed SRP RNA (7SL in higher eukaryotes, scR1 in yeast) and six proteins (SRP9, 14, 19, 54, 68 and 72 in higher eukaryotes, Srp21, 14, 54, 68, 72 and Sec65 in yeast, with Srp21 and Sec65 being homologues of higher eukaryotic SRP9 and SRP19, respectively). Srp54 is exclusively cytoplasmic, but other SRP protein subunits are detected in the nucleolus, suggesting that SRP assembly is largely nucleolar (43–45). Nuclear export of the assembled complex requires exportin 1 (Crm1 in yeast) (43–44,46).

SRP RNA is not extensively processed from its primary transcript. In HeLa cells, the 3′ terminal U-tract of 7SL is trimmed by up to 3 nt. and a single adenosine is added to the majority of the RNA (47). Using an *in vitro* adenylation assay, Perumal et al. (48) purified poly(A) polymerase gamma as an enzyme that can adenylate 7SL RNA. In contrast to 7SL, yeast scR1 predominantly ends in a 4–5 nt. U-tract (U_4–5_) and only a small fraction (2–3%) of the RNA is monoadenylated (49). As the 3′ U-tract is retained on scR1, Lhp1 is presumably actively displaced from the RNA during SRP biogenesis. Since Lhp1 is nuclear in yeast, this is likely to be necessary for export of the RNP to the cytoplasm. Several observations suggest that the TRAMP and exosome complexes also play roles in scR1 metabolism. Cells lacking Trf4 or Rrp44 activity contain a small amount of a truncated scR1 (50,51), and a nucleolar pool of scR1 accumulates in strains carrying the *rrp44–1* conditional mutation (44,52). Moreover, scR1 can be cross-linked to Trf4, Rrp6 and Rrp44 using ultraviolet light, indicating direct contacts between these proteins and the RNA (51,53–54). Additionally, the Rex1 exonuclease has been suggested to initiate turnover of scR1, since the accumulation of truncated scR1 in cells lacking Trf4p is dependent on Rex1 (50).

Here, we report that scR1, like many other stable RNAs, is aberrantly processed in the absence of Lhp1. We explore the requirements for the TRAMP and exosome complexes for correct localization and degradation of scR1 and, surprisingly, report that both TRAMP and exosome complexes are important to maintain the intact 3′ end of scR1. Cumulatively our data are consistent with the model that TRAMP and the exosome play central roles in SRP RNA metabolism, promoting assembly of the complex, but also directing degradation of the RNA when it fails quality control.

## MATERIAL AND METHODS

### Yeast strains and plasmids

Yeast strains (Supplementary Table S1) were grown in rich (YPD) or synthetic dropout media unless otherwise indicated. Gene deletion, tagging and promoter replacement were achieved using standard methods (55,56). Where proteins were depleted by repression of *GAL*-promoter alleles, cells were harvested for analysis of scR1 at the time at which growth rate in glucose-containing media deviated from logarithmic. Details of plasmid construction can be found in Supplementary Methods. *RRP44* and *TRF4* plasmids were as in (8,51) and (13), respectively.

### Protein and RNA analysis

RNA isolation, blotting and probing (57,58) used oligonucleotides complementary to scR1 nt. 11–31, 5S nt. 65–80, 5.8S nt. 31–47 and 5.8S precursors nt. 149–158 of mature 5.8S plus 8 downstream nt. Images were gathered using a Typhoon Trio scanner with ImageQuant software (GE Healthcare), and processed and labeled in Photoshop and Illustrator (Adobe). Ligation-mediated RT-PCR amplification of scR1 from RNA was as described (57). Briefly, a 3′ cordycepin-modified oligonucleotide (5′-GAACATTTTTTGGTTTAAACTAATTAACCGTCCC-3'dA or 5′-GATCTAGAGGATGGATATGGTGTTCAGG-3′dA) was ligated to the 3′ end of RNA. Reverse transcription was then primed with tag-rev (5′- TTCCCGGGACGGTTAATTAGTTTAAACC) or pJET1_fwd (5′-GCCTGAACACCATATCCATCC). Oligos Scer-cla233–250 (5′-CGATCTTTGCGGGCAGCC) and tag-rev or pJET1_fwd then primed amplification of scR1-specific products. Expression of Lhp1 was verified by western blot using an affinity-purified rabbit polyclonal anti-Lhp1 antibody (a gift from S. Wolin) and a mouse monoclonal anti-PGK (Invitrogen).

### Microscopy

Fluorescence *in situ* hybridization (59), used oligonucleotides scr1A complementary to nt. 1–34, scR1D nt. 476–97 and U3A nt. 47–76, modified to contain single Cy3, or Alexa488 fluorophores at the 5′ and 3′ termini. DAPI staining of live cells was done by incubation with 1 mg/ml DAPI in culture for 30 min. Live cells were mounted on slides in low-melt agar. All images were obtained using a Zeiss Axiovert 200 microscope with Plan-Apochromat x100 1.4NA objective, Axiovision software and an Axiocam monochrome camera, and processed and labeled in Photoshop and Illustrator (Adobe).

### Crosslinking and analysis of cDNA (CRAC)

CRAC experiments were carried out previously. The Illumina datasets used here (51,54) are accessible through GEO with accession numbers GSE46742 and GSE40046. ScR1-specific reads were identified in the datasets by mapping to the yeast genomic sequence (*Saccharomyces* Genome Database) using Novoalign (Novocraft). The distribution of reads along the *SCR1* genomic sequence was plotted as in (51). To identify oligoA-tailed sequences, reads were mapped using blastn, and those with ‘nonencoded tails’ extracted, i.e. the fragments of reads located between the genomic sequence and the trimmed 3′ end. The ‘non-encoded A-tails’ were identified as those nonencoded tails with at least 2 A's and not more than 20% non-A nucleotides. A more detailed description of the bioinformatics analysis can be found in (51,53).

## RESULTS

### Nuclear RNA surveillance machinery drives SRP RNA turnover

**Figure 1.**
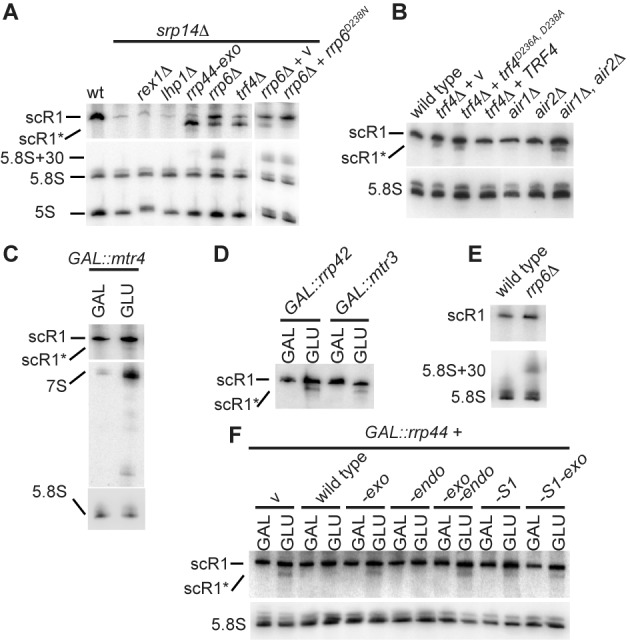
Mutations in TRAMP and exosome components lead to accumulation of truncated scR1. **(A–F)** Total RNA was extracted from the indicated strains, resolved on 6% w/v polyacrylamide 8M urea gels, and subjected to northern blotting using oligonucleotides directed against scR1, 5S, 5.8S and/or the 7S and 6S precursors to 5.8S. All cells were grown in media containing 2% w/v glucose (GLU), except strains containing GAL-promoter regulated alleles of *MTR4* or exosome components **(C, D, E)** which were grown in media containing 2% w/v each of raffinose and galactose (GAL) and shifted to GLU media to deplete the wild-type protein as described (Materials and Methods). **(B)** The *trf4Δ* strain was transformed with plasmids containing the wild-type *TRF4*, the allele encoding the catalytically inactive D236A, D238A mutant (*trf4^D236A^*,^*D238A*^) or no insert (v). **(A and F)***rrp6Δ* and *rrp6^D238N^* cells accumulate 5.8S+30 (11). **(A)***rex1Δ* cells contain a 3' extended form of 5S RNA (61). **(C)** cells lacking Mtr4 activity accumulate 5.8S precursors and particularly 7S (16).

To facilitate identification of factors that mediate SRP quality control and scR1 turnover, this was examined in the ‘sensitized’ background of cells lacking Srp14. Srp14 is required for assembly of the RNP, and in its absence the RNA is unstable (60), potentially making the effects of mutations that impair scR1 degradation more readily detectable. The accumulation of scR1 was then assessed by northern blotting (Figure 1A) in *srp14Δ* strains, also carrying mutations in Trf4 (the oligo(A) polymerase component of the TRAMP complex), Rrp6 or Rrp44 (the nuclease components of the exosome complex), the 3′ exonuclease Rex1 or the 3′ U-tract binding protein Lhp1. As previously observed, scR1 levels were greatly reduced in the absence of Srp14, and this was unaltered in cells that additionally lacked either Lhp1 or Rex1. In contrast, scR1 was substantially stabilized in cells lacking Trf4, Rrp6 or expressing only Rrp44-exo, which specifically lacks exonuclease activity. A shorter species, designated scR1*, was detected in all three strains. ScR1* was shown to be 3′ truncated as it was not detected by a probe to the 3′ end of the RNA (data not shown), and was more prominent than full-length scR1 in *srp14Δ*, *trf4Δ* and *srp14Δ*, *rrp44-exo* cells. In contrast *srp14Δ*, *rrp6Δ* cells predominately accumulated full-length scR1. These observations indicate that Rrp6 plays the major role in the initial steps of scR1 degradation, with TRAMP and Rrp44 being more important following 3′ truncation of scR1 to scR1*.

The exosome comprises a noncatalytic, nine component core barrel structure, that channels substrates to the exonuclease active site of Rrp44 (reviewed in (62)). Rrp6 stimulates activity of exosome-bound Rrp44 independent of Rrp6 catalytic activity, and facilitates threading of substrates through the exosome core (63,64). To test whether Rrp6 may contribute to Rrp44-dependent degradation of scR1, it was examined in *srp14Δ* cells expressing catalytically inactive Rrp6^D238N^ (6) as the only version of the protein. In these cells, scR1 was again stabilized, but scR1* was barely detectable. This is consistent with Rrp6 facilitating degradation of scR1* by Rrp44.

Extending these results, the effects of loss of TRAMP and exosome activities on scR1 were analysed in the background of an intact SRP complex (Figure 1B–F). The major form of the TRAMP complex comprises the oligo(A) polymerase Trf4, one of two partially redundant RNA binding, Zn-knuckle proteins Air1 and Air2 and the essential DEAV box RNA helicase Mtr4. As previously reported (50), scR1* was detected in RNA extracted from cells lacking Trf4. Cells expressing only the catalytically inactive Trf4^D236A, D238A^ protein (14,65) also accumulated scR1*, showing the oligo(A) polymerase activity of Trf4 to be required for efficient degradation of scR1. In addition, scR1* was detected in RNA extracted from cells lacking both Air1 and Air2, but not in either single mutant (Figure 1B) and also, faintly, in cells depleted of Mtr4 (Figure 1C).

Strains in which Rrp44 or the core exosome components Rrp42, Mtr3 or Rrp41 were genetically depleted (GLU lanes in Figure 1D and F) accumulated scR1*, confirming the importance of the exosome for efficient scR1 turnover (Figure 1D and F and data not shown). ScR1* was not seen in cells lacking Rrp6 (Figure 1E). These results are consistent with the analyses in *srp14Δ* cells, and specifically the conclusion that Rrp6 acts in the initial scR1 degradation step, prior to the TRAMP and exosome complexes.

Rrp44 has endonuclease, 3′-exonuclease and S1 RNA-binding domains that can be individually inactivated by mutations. The requirements for Rrp44 activities in scR1 degradation was assessed by expression of intact or mutant Rrp44 proteins from plasmids in strains depleted for endogenous Rrp44 (Figure 1F). Loss of endonuclease activity did not yield accumulation of scR1*, but this was observed in strains lacking only the exonuclease or both activities. We conclude that the Rrp44 exonuclease active site processes scR1*, and that endonuclease activity is not required for generation of scR1*, consistent with previous analysis (51). A mutation in the S1 RNA binding domain (G916E) that reduces *in vitro* RNA binding (3) led to detectable scR1* accumulation. We conclude that the S1 domain of Rrp44 contributes to its activity in degrading scR1. This is consistent with recent structural data indicating that the S1 domain is close to the pathway to the Rrp44 exonuclease active site that is followed by RNA substrates with short single stranded tails, such as scR1 (62,66).

### Lhp1 maintains the 3′ end integrity of scR1

**Figure 2.**
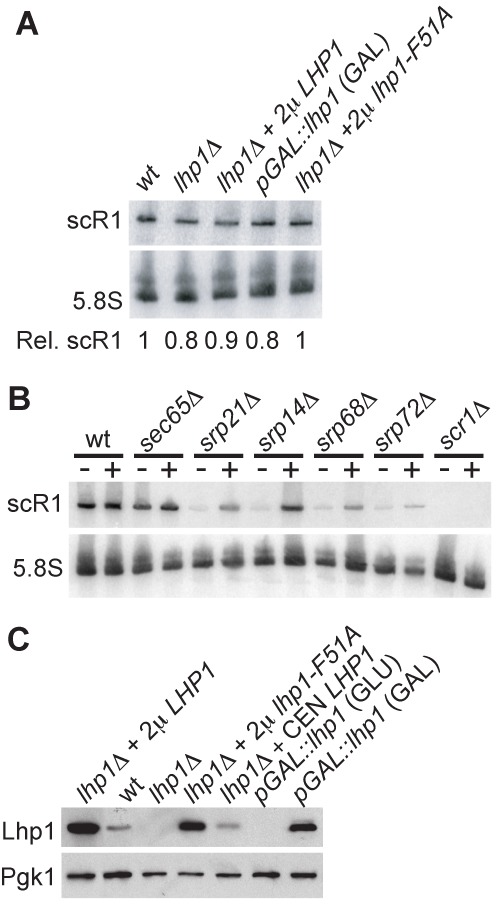
Altering Lhp1p expression affects scR1 levels in cells deficient in SRP core proteins, but not wild-type cells. **(A, B)** Total RNA was extracted from the indicated strains and analysed as in Figure 1. **(A)** Strains were either wild-type (wt), lacked the genomic copy of *LHP1 (lhp1Δ*) and contained the plasmids indicated or contained a GAL-promoter-driven allele of *LHP1*. Cells were grown in GLU media, except those containing the *GAL::lhp1* allele which were grown in GAL media. **(B)** Strains were wt or carried individual deletions of gene encoding protein components of SRP. Each strain was transformed with either a high copy (2μ) empty vector (−) or the same plasmid containing *LHP1* (+). **(C)** Cell lysates were resolved on an SDS-PAGE gel, blotted to nitrocellulose membrane and probed with antibodies against Lhp1 and Pgk1 (loading control). Strains and growth conditions were as in **(A)** except that the *lhp1Δ* strain was additionally transformed with a low copy (CEN) plasmid containing Lhp1, and the *GAL::lhp1* strain was grown in both GAL and GLU media.

Lhp1 binds the oligo(U) tract present at the 3′ ends of all Pol III primary transcripts, including scR1, and is required for correct processing and/or stability of many RNA species (23,25,38,67). Despite this, scR1 levels were unchanged in *srp14Δ* cells in the presence or absence of Lhp1 (Figure 1A), prompting further analysis of the role of Lhp1 in scR1 metabolism. In otherwise wild-type cells, neither the absence of Lhp1 nor its over-expression clearly affect scR1 levels (Figure 2A) (24,50). However, over-expression of Lhp1 did result in increased scR1 abundance in cells lacking Srp14 or other core SRP proteins also required for stable assembly of the RNP (Figure 2B). This is consistent with elevated levels of Lhp1 protecting scR1 from degradation when it is unable to assemble into SRP.

**Figure 3.**
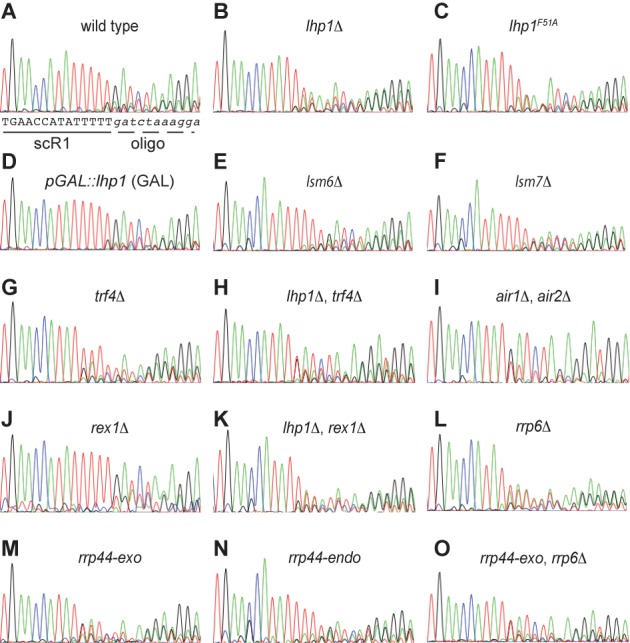
The scR1 3' end is altered in the absence of Lhp1 and in TRAMP and exosome mutants. RT-PCR products covering the 3' portion of scR1 were generated from total RNA extracted from the indicated strains and sequenced directly (Materials and Methods). The portion of the trace file corresponding to the 3' end of scR1 and the junction with the oligonucleotide ligated to the RNA (5'-GATCTAGAGGATGGATATGGTGTTCAGG-3'dA in all cases) are shown for each strain. Interpretation of the sequence of scR1 is shown below the trace for the wild-type strain **(A)**. Trace files are displayed in standard ABI chromatogram colours, images were captured using Grab (Apple) from sequence files displayed using 4Peaks (Mekentosj).

To investigate whether the absence of Lhp1 affects the processing of scR1, the 3′ end sequence of the RNA was characterized by oligonucleotide ligation and RT-PCR (Materials and Methods) (57,68), followed by sequencing of resulting PCR products, yielding information on the total pool of amplified scR1 (Figure 3). Cloned amplification products were also sequenced to provide a sampling of individual scR1 3′ ends (Supplementary Table S2). ScR1 has a 3′ terminal U_4–5_ tract (44,69) and this was unchanged on over-expression of Lhp1 (Compare Figure 3A and D). In contrast, sequencing RT-PCR products amplified from cells lacking Lhp1 indicated that the majority of the RNA terminated in 2 U's, with the following positions containing a mixture of U and A (Figure 3B). The presence of non-templated A residues was confirmed by sequencing cloned PCR products. Of 24 clones, only 1 had a sequence indicating a wild-type 3′ end on the RNA (U_4_), the remainder being shortened and/or containing 1–4 non-templated A residues. In contrast, 17 of 20 individually sequenced cloned PCR products from wild-type cells had sequences indicating that the RNA terminated in U_4_ or U_5_.

To relate integrity of the 3′ end of scR1 to activity of Lhp1, rather than just its presence, the 3′ end of scR1 was determined in cells expressing only Lhp1 with a F51A mutation. Lhp1^F51A^ corresponds to human La^F35A^, which abolishes 3′ U-tract binding (22). Lhp1^F51A^ cells contained trimmed, adenylated scR1 similar to cells lacking Lhp1 (Figure 3C). From these data we conclude that binding of Lhp1 to scR1 protects the 3′ end of the RNA. In the absence of Lhp1 both exonuclease and adenyltransferase activities act on scR1.

### The TRAMP and exosome complexes maintain scR1 3′ end integrity

**Figure 4.**
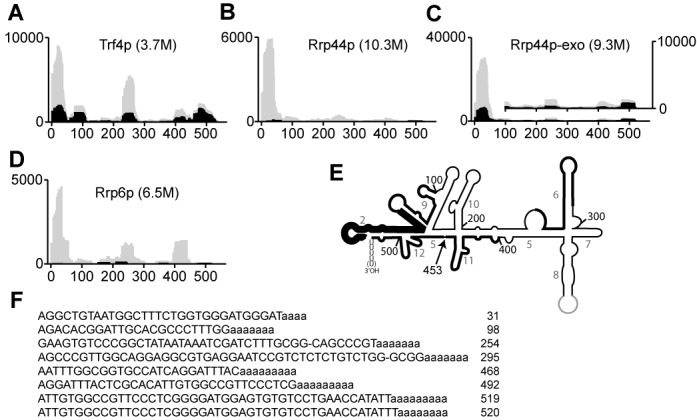
Trf4, Rrp6 and Rrp44 associate with scR1. Sequence reads mapped to scR1 were extracted from datasets from CRAC experiments (Materials and Methods) carried out with HTP-tagged Trf4 **(A)**, Rrp44 **(B)**, Rrp44-exo **(C)** or Rrp6 **(D)**, and plotted as histograms representing hits density per million sequences aligned to the *Saccharomyces cerevisiae* genome. Gray shading denotes total scR1 sequences, whereas black shading is the subset of these that contain two or more nonencoded adenosines at the 3' end. Note that the Y-axis scale is substantially different in panel C compared to the others, since the Rrp44-exo dataset contained proportionally more hits to scR1. The inset has an expanded Y-axis scale to more clearly show the occurrence of 3' oligoadenylated sequences outside of the 5' region. **(E)** A line diagram of the secondary structure of scR1, with the main peaks of sequences mapped to it in the Trf4p dataset highlighted by thickening of the line, which is greater in the 5' portion of the RNA to reflect the greater number of sequences mapping to it. The position of nucleotide 453, the likely end point of scR1* is indicated and helices are numbered as in (55). **(F)** Representative, abundant sequences from the Trf4 dataset that map to different regions of the RNA are shown. The number to the right of each sequence is the final nt of scR1 present in it. The third and fourth sequences, in common with a number of others in the dataset, are missing a base when compared to genomic *SCR1* (indicated ‘−‘). This may be a cross-linking induced error, as previously observed (70).

The above data, combined with the observation that the TRAMP and exosome complexes play roles in scR1 turnover (Figure 1), prompted examination of the 3′ end of scR1 in cells lacking activities of these complexes (Figure 3 and Supplementary Table S2). In cells lacking the TRAMP poly(A) polymerase Trf4, sequencing of total RT-PCR products revealed the presence of a U-tract at the 3′ end of scR1, as well as evidence for some shortened species (Figure 3G). This was confirmed by sequencing individual, cloned fragments, which were more heterogeneous than in wild-type cells. In addition, clones were identified that were significantly truncated. Several ended at nucleotide 453, which lies within core helix 5 of scR1, just 3′ to the junction of helices 5, 10 and 11 (57; and see Figure 4E). We predict that this corresponds to the truncated scR1* species seen in northern analyses (Figure 1). ScR1 RT-PCR products amplified from cells lacking both Lhp1 and Trf4, revealed a trimmed 3′ terminal U-tract similar to *lhp1Δ* cells, but no clear indication of adenylation (Figure 3H). This was confirmed by sequencing of individually cloned PCR products; only 1 of 18 clones extending to the 3′ end of scR1 was adenylated (Supplementary Table S2). This residual adenylation of scR1 in *lhp1Δ*, *trf4Δ* cells is likely to involve Trf5, which is homologous to and semi-redundant with Trf4. We conclude that Trf4 is responsible for the major adenylation activity on scR1 in *lhp1Δ* cells.

The heterogeneity of the 3′ end of scR1 in *trf4Δ* cells suggested that association of TRAMP with the RNA has an unexpected, positive role in maintaining its integrity. This model was supported by sequencing of the 3′ end of scR1 in cells lacking the redundant Air1 and Air2 proteins, which are present in TRAMP complexes containing either Trf4 or Trf5. The majority of scR1 clones recovered from the *air1Δ, air2Δ* strain carried a single 3′ terminal U residue (Figure 3I and Supplementary Table S2). Thus, in addition to its importance for scR1 turnover, TRAMP is necessary to maintain the 3′ integrity of scR1. To determine whether deficiencies in exosome activities affected the integrity of the 3′ end of scR1, this was examined in cells lacking Rrp6, or expressing either Rrp44-endo or Rrp44–exo as the only form of Rrp44 (Figure 3L–N and Supplementary Table S2). In cells expressing Rrp44-endo, scR1 had a wild-type U_4–5_ 3′ end. However, scR1 was truncated in cells expressing Rrp44-exo or lacking Rrp6. In both cases sequencing of individual cloned RT-PCR products revealed that a fraction of the RNA was adenylated in addition to being shortened. We conclude that the TRAMP and exosome complexes play a direct and positive role in protecting the RNA from aberrant processing. Only a small fraction of scR1 molecules retain the normal 3′ end in cells lacking the activities of these complexes.

In a strain lacking Rrp6 and expressing only Rrp44-exo, the truncation of scR1 was similar to that seen in the individual mutants, indicating that a nonexosomal nuclease(s) is responsible for trimming the RNA in these cells (Figure 3O). A candidate for such a nuclease is Rex1 (50). In *rex1Δ* cells, the sequence of scR1 RT-PCR products was broadly similar to the wild-type, but there were consistent indications of an increase in the RNA population extending to U_5_ (Figure 3J). Sequencing individual cloned RT-PCR products confirmed this, and also identified RNAs extending to U_6_ (Supplementary Table S2). These data suggest that scR1 transcription extends, at least in some cases, to the end of the T_6_-tract that forms the *SCR1* pol III transcription termination signal, with Rex1 removing 1 or 2 nucleotides from the terminal U-tract of the RNA. Contrasting with this, we found no evidence for Rex1 being solely responsible for trimming scR1 in the absence of Lhp1. The 3′ end of scR1 in *lhp1Δ*, *rex1Δ* cells was similar to that in *lhp1Δ* cells, with the RNA both truncated and adenylated (Figure 3K and Supplementary Table S2).

### Interactions of TRAMP and exosome components with scR1

The preceding data indicate that both efficient turnover of scR1 and maintenance of the 3′ sequence require TRAMP and exosome activities. Direct association of RNAs with Trf4 and exosome components has recently been examined using the CRAC (cross-linking and analysis of cDNAs) method (70,71). ScR1 sequences are present in published high-throughput datasets generated from strains expressing the protein of interest HTP-tagged to allow its purification, but otherwise wild-type (51,54), and analyses of these data revealed that, in each case, the greatest number of scR1 sequences corresponded to the 5′ end of the RNA, within the first 50 nt. (Figure 4A–D). In the scR1 secondary structure the 5′ and 3′ ends of the RNA are brought together, and cross-linking to the 5′ end could reflect activity toward the 3′ end (Figure 4E). The Trf4 CRAC dataset contained the largest proportion of scR1 sequences that did not map to the 5′ end, demonstrating that Trf4 associates with multiple regions of scR1 (Figure 4A).

Some scR1 sequences in CRAC datasets contained nonencoded adenosines at their 3′ end (Figure 4A–D, regions of graphs shaded in black), indicative of TRAMP activity on the RNA. The proportion of 3′-oligoadenylated sequences was highest in association with Trf4, and these were particularly prominent in 3′ end sequences of the RNA. Representative examples of oligoadenylated sequences from the Trf4 dataset are shown (Figure 4F). These findings are further evidence for Trf4 having a key, direct role in surveillance and turnover of scR1. The proportion of hits within scR1, and the fraction of these that were 3′-oligoadenylated, were both increased in the Rrp44-exo dataset when compared to that obtained with wild-type Rrp44 (compare Figure 4B and C). This confirms the importance of the exonuclease function of Rrp44 in scR1 turnover.

### Over-expression of Lhp1p leads to nuclear retention of scR1

**Figure 5.**
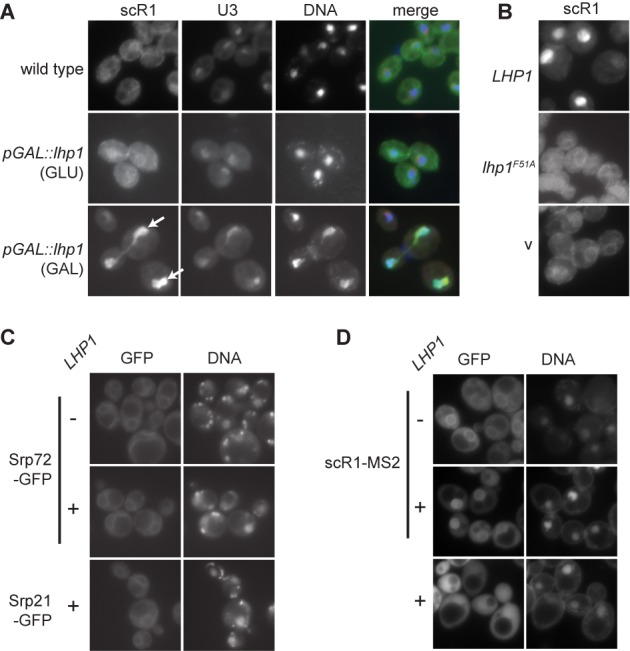
ScR1, but not SRP proteins, accumulate in the nucleolus of cells over-expressing Lhp1. **(A)** Fluorescence *in situ* hybridization (FISH) was carried out on wild-type cells or cells containing the *pGAL::lhp1* allele grown in the indicated media using fluorescently labeled probes scR1A and U3A (Materials and Methods). DNA was stained with DAPI. Individual channels and merged (scR1 in green; U3 snoRNA red; DNA blue) images are shown as indicated. Nucleolar scR1 in cells over-expressing Lhp1 is indicated with arrows. **(B)** FISH was carried out as in **(A)** using the scR1A probe but on wild-type cells transformed with high copy (2μ) plasmids containing the wild-type or F51A versions of LHP1 as indicated or an empty vector control (v). **(C)** Yeast expressing an MS2 phage coat protein-GFP fusion protein, scR1 modified to contain tandem copies of the MS2-binding site (scR1-MS2) and **(D)** yeast strains expressing genomically GFP tagged SRP proteins as indicated and/or containing a high copy plasmid without (−) or with (+) *LHP1* as indicated were stained with DAPI. **(A–D)** Fluorescent images of cells were captured as described (Materials and Methods).

The effects of altering Lhp1 levels on scR1 localization were examined using fluorescence *in situ* hybridization (FISH). In wild-type cells scR1 is largely cytoplasmic, with stronger signals at the nuclear envelope and cell periphery, consistent with the role of SRP in protein targeting to the endoplasmic reticulum (Figure 5A) (43,44). ScR1 localization was not appreciably altered in the absence of Lhp1. Thus, although Lhp1p is required to maintain 3′ integrity of scR1, it is dispensable for its correct localization and, by inference, SRP assembly. In contrast, scR1 accumulated within the nucleus when Lhp1 was over-expressed with the strongest signal in the nucleolus, co-localizing with the U3 snoRNA (Figure 5A, B). ScR1 localization was not altered when Lhp1^F51A^ was over-expressed, consistent with the mutant protein failing to bind scR1 (Figure 5B).

To ascertain whether Lhp1 over-expression also results in the nuclear accumulation of SRP proteins, the localization of GFP-tagged Srp21 and Srp72 was examined. These proteins assemble with scR1 in the nucleus (43,44). Srp21 and Srp72 were predominantly cytoplasmic and concentrated at the endoplasmic reticulum in wild-type cells, and there was no change to this pattern when Lhp1 was over-expressed (Figure 5C and data not shown). For comparison, the localization of scR1 was visualized by expression of scR1-MS2, which contains binding sites for the MS2 phage protein, together with a GFP-MS2 fusion (Figure 5D). MS2-GFP was enriched in the nucleus when Lhp1 was over-expressed in cells expressing the scR1-MS2, consistent with the results of scR1 FISH analyses (Figure 5A, B). Since scR1 levels do not significantly change on over-expression of Lhp1, these results suggest that excess Lhp1 delays SRP assembly.

### Lack of Lsm proteins does not affect scR1 localization or integrity

The association of Lhp1 with scR1 and other pol III transcripts is compromised in the absence of an intact Lsm complex (38). Cells lacking individual Lsm proteins were therefore examined for scR1 3′ end integrity, localization and the effects of over-expression of Lhp1. The localization of scR1 was unaltered in cells lacking any of the nonessential proteins Lsm4, 5, 6, 7. Over-expression of Lhp1 in these strains induced the nuclear accumulation of scR1, to an extent similar to that seen in the wild-type (Supplementary Figure S1). This strongly indicates that Lhp1 is able to bind to scR1 in the absence of an intact Lsm complex. 3′ end sequencing of scR1 in cells lacking Lsm4, 5, 6 or 7, indicated that the RNA had the wild-type U_4–5_ 3′ terminus (Figure 3E, F and data not shown). Overall the data indicate that the biogenesis of scR1 and SRP are not strongly affected by the absence of an intact Lsm complex.

### Nucleolar retention of scR1 in cells deficient in TRAMP and exosome activities

**Figure 6.**
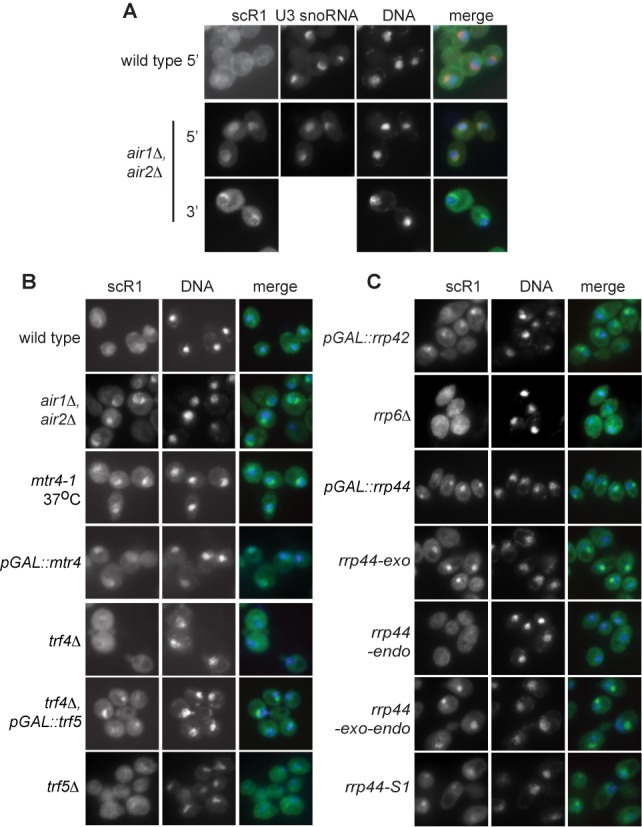
ScR1 accumulates in the nucleolus of cells lacking TRAMP or exosome activities. Yeast strains as indicated were analysed by FISH, DNA stained with DAPI and images captured as in Figure 4, except for *air1Δ*, *air2Δ* cells where an additional probe, scR1D, complementary to the 3' portion of scR1 was used. Cells were grown at 30°C, except thermosensitive *mtr4–1* cells which were shifted from 25 to 37°C for 1 h prior to fixing. Strains containing GAL-promoter regulated alleles were treated as in Figure 1 prior to fixing.

ScR1 localization was examined by FISH in strains compromised for TRAMP and nuclear exosome activities (Figure 5). Strong accumulation of scR1 adjacent to nuclear DNA was seen in the *air1Δ, air2Δ* double mutant, but not *air1Δ* or *air2Δ* single mutant cells (Figure 6A, B and data not shown). The signal corresponded to the nucleolus since it co-localized with U3 snoRNA, and it was seen with probes specific to both 5′ (nts. 1–34) and 3′ (nts. 476–497) regions of scR1 (Figure 6A). This indicates that this signal is not generated by scR1*, or other fragments generated through degradation of the RNA. Nucleolar accumulation of scR1 was also seen on depletion of the Mtr4 helicase component of the TRAMP complex, and in *mtr4–1* cells incubated at the nonpermissive temperature (Figure 6B). In contrast, scR1 localization was unperturbed in *trf4Δ* cells, and this was also the case in *trf5Δ* cells (Figure 6B). However, depletion of Trf5 from *trf4Δ* cells led to nucleolar accumulation of scR1. Together these date indicate that an intact TRAMP complex, containing either Trf4 or Trf5 and either Air1 or Air2, is necessary for correct localization of scR1.

Lack of Rrp6 did not alter scR1 localization, whereas depletion of Rrp44 or the core exosome components Mtr3 or Rrp42 led to nucleolar accumulation of the RNA (Figure 6C and data not shown). ScR1 also accumulated in the nucleus of cells expressing variants of Rrp44 lacking either the exonuclease or S1 RNA binding activities as the only form of the protein. Thus an intact exosome and the activities of Rrp44 (though not Rrp6) are also necessary for correct scR1 localization.

## DISCUSSION

**Figure 7.**
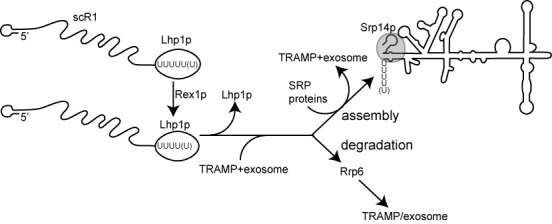
Interactions of Lhp1 and RNA surveillance factors with scR1. A model integrating findings presented herein into a biogenesis and quality control pathway for scR1/SRP. The primary scR1 transcript contains a terminal U_5/6_ tract and is bound by Lhp1. Rex1 competes with Lhp1 for binding and trims the RNA by 1–2 nucleotides to the mature form of the RNA. Lhp1 is displaced from the RNA and both the TRAMP and exosome complexes associate with it. Correct folding/association with SRP proteins leads to completion of biogenesis of SRP, while any RNA that is not assembled into the complex is degraded, with Rrp6 acting prior to the exosome complex.

Assembly of RNPs, with associated RNA processing and quality control, is a considerable task. The activities of the ubiquitous and abundant SRP complex have been extensively studied (41,42). However, although an outline biogenesis pathway for SRP has been established, little is known about factors involved, its regulation or quality control. From the data presented here, a model of SRP biogenesis and quality control can be proposed that incorporates roles for Lhp1, Rex1 and the nuclear RNA surveillance machinery (Figure 7).

The RNA pol III transcription termination signal of *SCR1* is T_6_, and the mature RNA has a terminal U-tract comprising U_4–5_. Cells lacking Rex1 contain scR1 ending almost entirely in U_5_ or U_6_, strongly suggesting that the primary scR1 transcript normally extends to U_5_ or U_6_, with Rex1 removing 1–2 nucleotides. Rex1 also processes of a number of other non-coding RNAs, including the RNA pol III-transcribed 5S rRNA and tRNAs (50,61,72).

Analyses in cells that lack Srp14 and are unable to stably assemble SRP complexes identified a role for Rrp6 early in the degradation of misassembled scR1 (Figure 1A). Downstream of Rrp6, the TRAMP complex and the exonuclease activity of Rrp44 have more important roles in turnover of misassembled scR1, and a 3′ truncated species (scR1*) was present in cells lacking activities of either of these complex, regardless of whether or not Srp14 was present. The 3′ end of the scR1* fragment, probably located at nt. 453, appears to define a point in the degradation of the RNA at which TRAMP and exosome activities are particularly important. Efficient degradation from this point requires the poly(A) polymerase activity of Trf4 (Figure 1B), which is notable as catalytically inactive Trf4^D236A, D238A^ can still direct turnover of most of the RNAs that it targets (3,73–74).

Recovery of scR1 fragments with Trf4, Rrp44 and Rrp6 in UV-crosslinking (CRAC) experiments revealed direct association of these factors with scR1 and provided insights into where each protein binds the RNA (Figure 4) (51,54). These experiments were carried out in wild-type cell backgrounds, and therefore not influenced by alterations to other RNA binding/processing factors or SRP proteins. The predominance of 5′ end-derived scR1 sequences in each CRAC dataset may reflect association of TRAMP and exosome with short scR1 fragments in late stages of 3′-5′ degradation. This is clearly the case for fragments carrying 3′-oligoadenylation, which are prominent in the Trf4 dataset. However, the secondary structure of scR1 brings the 5′ and 3′ ends of the RNA close together in the Alu-domain (Figure 4E) (57,58). Cross-linking of Trf4p, Rrp44p and Rrp6p to 5′ regions of the RNA might therefore reflect ‘docking’ of these proteins on the scR1 structure prior to and/or during their activities at the 3′ end of the RNA. The Alu-domain in scR1 is bound by an Srp14 dimer, which restructures the 5′ and 3′ regions of the RNA (75,76) and seems very likely to be mutually exclusive with binding of the surveillance machinery. Binding of Srp14 may therefore be a key step in determining the fate of nascent SRP RNA. The nuclease defective Rrp44p-exo protein was more frequently associated with scR1 than was wild-type Rrp44, and these fragments were more frequently oligoadenylated. This suggests that the absence of the Rrp44 exonuclease activity leads to prolonged association of the exosome with scR1 molecules that have been targeted for degradation. The small number of oligoadenylated scR1 fragments recovered with wild-type Rrp44p presumably reflects their efficient removal by the nuclease activity of the enzyme.

Multiple oligoadenylated fragments derived from the 3' end of scR1 were present in the Trf4 CRAC datasets (Figure 4). Many of these sequences contained three or fewer U residues, whereas only 2.2% contained four or five residues, strongly indicating that Trf4 specifically associates with scR1 that has already been truncated. Rrp6 functions early in scR1 degradation (Figure 1A), and trimming of the RNA by Rrp6 may be a key step that then allows Trf4 to act on scR1. A short U-tract would reduce affinity for Lhp1, and this may also be an important selection criterion for degradation of the RNA. The truncated scR1* species was absent from cells lacking both Trf4 and Rex1 (data not shown and (50)), suggesting that Rex1 functions upstream of Trf4 in scR1 degradation. However, in cells unable to assemble SRP through lack of Srp14, loss of Rex1 did not affect turnover of scR1 (Figure 1A). We therefore propose that the major pathway that initiates scR1 degradation is via Rrp6 followed by TRAMP and exosome. An alternative, Rex1-dependent pathway may initiate degradation of scR1 in the absence of TRAMP/exosome activity, but stalls at scR1*.

Similar to its role in orchestrating 3' processing of other non-coding RNAs, Lhp1 protects the correct 3' end of scR1. In the absence of Lhp1p, the 3' end of scR1 is exposed to nuclease activity, with consequent trimming leaving U_2–3_, and a substantial fraction of the RNA becomes oligoadenylated by Trf4. The identity of the nuclease(s) that trims scR1 in the absence of Lhp1 remains unclear, but it is not primarily Rex1, as the 3' end of scR1 was similar in *lhp1Δ* single and *lhp1Δ rex1Δ* double mutant strains. As both Rrp6 and Rrp44 are required to maintain the correct scR1 3' end in the presence of Lhp1, it was not possible to ascertain whether these nucleases were responsible for trimming scR1 in the absence of Lhp1. The alterations to the 3' end of scR1 that occur in *lhp1Δ* cells do not significantly hinder SRP assembly or nuclear export, as the amount and localization of scR1 in cells is unaffected in the absence of Lhp1 (Figures 2A and 5A). Finding that lack of individual Lsm proteins did not affect scR1 integrity or localization was somewhat surprising given previous observations that Lsm proteins are required for efficient association of Lhp1 with scR1 and other pol III RNAs, as well as normal precursor tRNA processing (38). Association of Lhp1 with RNAs in this previous study was carried out using a protein A-tagged version of the protein. ScR1 3' integrity is partially compromised in strains expressing tagged Lhp1 (JDB unpublished), and it may be that a wild-type Lhp1 is more able to bind to scR1 in the absence of an intact Lsm complex than the tagged protein.

Over-expression of Lhp1 both stabilizes scR1 when it cannot be assembled into SRP (Figure 2B), and leads to significant accumulation of the RNA in the nucleus, and particularly nucleolus, of otherwise wild-type cells (Figure 5A, B, D). The SRP proteins examined, Srp72 and Srp21, did not accumulate with scR1 in the nucleus when Lhp1 is over-expressed (Figure 5C). Since Srp72 and Srp21 form tight complexes with Srp68 and Srp14, respectively, it is unlikely that any core SRP proteins are bound to this pool of nuclear-retained scR1. This suggests that scR1 accumulated in the nucleus on Lhp1 over-expression is RNA that would normally be turned over but which is protected by prolonged Lhp1 binding, as in *srp14Δ* cells. Consistent with Lhp1 competing with surveillance factors for scR1 turnover, extra copies of *LHP1* suppressed the appearance of scR1* in *trf4Δ* cells (50). However, over-expression of Lhp1 did not lead to an appreciable increase in full-length scR1 levels in wild-type cells, and Lhp1 may also compete with and inhibit binding of SRP proteins and assembly factors.

A complete TRAMP complex is required for correct localization of scR1, and removal of Mtr4, Air1 and Air2, or Trf4 and Trf5 resulted in nucleolar accumulation of scR1. That lack of Trf4 alone leads to the presence of scR1* in cells, but not accumulation of scR1 in the nucleolus, argues against the conclusion that nucleolar scR1 in TRAMP-deficient cells is only RNA that is on the degradation pathway. Further, a probe directed to the 3' region of scR1 detected the RNA in the nucleolus of *air1Δ air2Δ* cells, excluding the possibility that this pool of RNA represented only partial degradation products (Figure 5A). These data lead us to propose that binding of an intact TRAMP complex (containing either Trf4 or Trf5) is a requisite step in efficient SRP biogenesis. Cells lacking TRAMP components contain scR1 with an aberrantly trimmed 3' end, indicating that, like Lhp1, TRAMP acts to protect the 3' end of scR1. More unexpectedly, the exosomal nucleases Rrp6 and Rrp44 also apparently provide protection to scR1, with the RNA being aberrantly processed in cells lacking catalytic activities of either exonuclease. Lack of catalytic activity of these proteins would not be expected to lead directly to loss of a protective function on scR1. However, an indirect effect could be via the reduction of free exosome complexes in the nucleus available to bind scR1 due to its increased association with RNAs that it is unable to efficiently degrade. As with TRAMP, the exosome is also necessary for correct scR1 localization.

Overall, the data presented here lead us to propose that newly transcribed scR1 is initially bound by Lhp1 and then associates with the TRAMP and exosome complexes. This ordered series of events protects scR1 from nuclease activities that would otherwise aberrantly trim the RNA. Further, this may facilitate the decision between biogenesis of SRP and a discard pathway for exosome-dependent degradation of scR1.

## SUPPLEMENTARY DATA

Supplementary Data are available at NAR Online.

SUPPLEMENTARY DATA

## References

[1] Lebreton A., Seraphin B. (2008). Exosome-mediated quality control: substrate recruitment and molecular activity. Biochim. Biophys. Acta.

[2] Houseley J., Tollervey D. (2009). The many pathways of RNA degradation. Cell.

[3] Schneider C., Anderson J.T., Tollervey D. (2007). The exosome subunit Rrp44 plays a direct role in RNA substrate recognition. Mol. Cell.

[4] Dziembowski A., Lorentzen E., Conti E., Seraphin B. (2007). A single subunit, Dis3, is essentially responsible for yeast exosome core activity. Nat. Struct. Mol. Biol..

[5] Liu Q., Greimann J.C., Lima C.D. (2006). Reconstitution, activities, and structure of the eukaryotic RNA exosome. Cell.

[6] Burkard K.T., Butler J.S. (2000). A nuclear 3'-5' exonuclease involved in mRNA degradation interacts with Poly(A) polymerase and the hnRNA protein Npl3p. Mol. Cell. Biol..

[7] Lebreton A., Tomecki R., Dziembowski A., Seraphin B. (2008). Endonucleolytic RNA cleavage by a eukaryotic exosome. Nature.

[8] Schneider C., Leung E., Brown J., Tollervey D. (2009). The N-terminal PIN domain of the exosome subunit Rrp44 harbors endonuclease activity and tethers Rrp44 to the yeast core exosome. Nucleic Acids Res..

[9] Schaeffer D., Tsanova B., Barbas A., Reis F.P., Dastidar E.G., Sanchez-Rotunno M., Arraiano C.M., van Hoof A. (2009). The exosome contains domains with specific endoribonuclease, exoribonuclease and cytoplasmic mRNA decay activities. Nat. Struct. Mol. Biol..

[10] Turk E.M., Das V., Seibert R.D., Andrulis E.D. (2013). The mitochondrial RNA landscape of *Saccharomyces cerevisiae*. PLoS One.

[11] Briggs M.W., Burkard K.T., Butler J.S. (1998). Rrp6p, the yeast homologue of the human PM-Scl 100-kDa autoantigen, is essential for efficient 5.8 S rRNA 3' end formation. J. Biol. Chem..

[12] Lacava J., Houseley J., Saveanu C., Petfalski E., Thompson E., Jacquier A., Tollervey D. (2005). RNA degradation by the exosome is promoted by a nuclear polyadenylation complex. Cell.

[13] Vanacova S., Wolf J., Martin G., Blank D., Dettwiler S., Friedlein A., Langen H., Keith G., Keller W. (2005). A new yeast poly(A) polymerase complex involved in RNA quality control. PLoS Biol..

[14] Wyers F., Rougemaille M., Badis G., Rousselle J.C., Dufour M.E., Boulay J., Regnault B., Devaux F., Namane A., Seraphin B. (2005). Cryptic pol II transcripts are degraded by a nuclear quality control pathway involving a new poly(A) polymerase. Cell.

[15] Houseley J., Tollervey D. (2006). Yeast Trf5p is a nuclear poly(A) polymerase. EMBO Rep.

[16] de la Cruz J., Kressler D., Tollervey D., Linder P. (1998). Dob1p (Mtr4p) is a putative ATP-dependent RNA helicase required for the 3' end formation of 5.8S rRNA in *Saccharomyces cerevisiae*. EMBO J..

[17] Jackson R.N., Klauer A.A., Hintze B.J., Robinson H., van Hoof A., Johnson S.J. (2010). The crystal structure of Mtr4 reveals a novel arch domain required for rRNA processing. EMBO J..

[18] Klauer A.A., van Hoof A. (2013). Genetic interactions suggest multiple distinct roles of the arch and core helicase domains of Mtr4 in Rrp6 and exosome function. Nucleic Acids Res..

[19] Stefano J.E. (1984). Purified lupus antigen La recognizes an oligouridylate stretch common to the 3' termini of RNA polymerase III transcripts. Cell.

[20] Mathews M.B., Francoeur A.M. (1984). La antigen recognizes and binds to the 3'-oligouridylate tail of a small RNA. Mol. Cell. Biol..

[21] Fan H., Goodier J.L., Chamberlain J.R., Engelke D.R., Maraia R.J. (1998). 5' processing of tRNA precursors can Be modulated by the human La antigen phosphoprotein. Mol Cell Biol.

[22] Teplova M., Yuan Y.R., Phan A.T., Malinina L., Ilin S., Teplov A., Patel D.J. (2006). Structural basis for recognition and sequestration of UUU(OH) 3' temini of nascent RNA polymerase III transcripts by La, a rheumatic disease autoantigen. Mol. Cell.

[23] Yoo C.J., Wolin S.L. (1997). The yeast La protein is required for the 3' endonucleolytic cleavage that matures tRNA precursors. Cell.

[24] Kufel J., Allmang C., Chanfreau G., Petfalski E., Lafontaine D.L., Tollervey D. (2000). Precursors to the U3 small nucleolar RNA lack small nucleolar RNP proteins but are stabilized by La binding. Mol. Cell. Biol..

[25] Xue D., Rubinson D.A., Pannone B.K., Yoo C.J., Wolin S.L. (2000). U snRNP assembly in yeast involves the La protein. EMBO J..

[26] Pannone B.K., Xue D., Wolin S.L. (1998). A role for the yeast La protein in U6 snRNP assembly: evidence that the La protein is a molecular chaperone for RNA polymerase III transcripts. EMBO J..

[27] Calvo O., Cuesta R., Anderson J., Gutierrez N., Garcia-Barrio M.T., Hinnebusch A.G., Tamame M. (1999). GCD14p, a repressor of GCN4 translation, cooperates with Gcd10p and Lhp1p in the maturation of initiator methionyl-tRNA in *Saccharomyces cerevisiae*. Mol. Cell. Biol..

[28] Chakshusmathi G., Kim S.D., Rubinson D.A., Wolin S.L. (2003). A La protein requirement for efficient pre-tRNA folding. EMBO J..

[29] Huang Y., Intine R.V., Mozlin A., Hasson S., Maraia R.J. (2005). Mutations in the RNA polymerase III subunit Rpc11p that decrease RNA 3' cleavage activity increase 3'-terminal oligo(U) length and La-dependent tRNA processing. Mol. Cell. Biol..

[30] Huang Y., Bayfield M.A., Intine R.V., Maraia R.J. (2006). Separate RNA-binding surfaces on the multifunctional La protein mediate distinguishable activities in tRNA maturation. Nat. Struct. Mol. Biol..

[31] Naeeni A.R., Conte M.R., Bayfield M.A. (2012). RNA chaperone activity of human La protein is mediated by variant RNA recognition motif. J. Biol. Chem..

[32] Simons F.H., Rutjes S.A., van Venrooij W.J., Pruijn G.J. (1996). The interactions with Ro60 and La differentially affect nuclear export of hY1 RNA. RNA.

[33] Grimm C., Lund E., Dahlberg J.E. (1997). In vivo selection of RNAs that localize in the nucleus. EMBO J..

[34] Boelens W.C., Palacios I., Mattaj I.W. (1995). Nuclear retention of RNA as a mechanism for localization. RNA.

[35] Intine R.V., Dundr M., Misteli T., Maraia R.J. (2002). Aberrant nuclear trafficking of La protein leads to disordered processing of associated precursor tRNAs. Mol. Cell.

[36] Fernandez C.F., Pannone B.K., Chen X., Fuchs G., Wolin S.L. (2004). An Lsm2-Lsm7 complex in *Saccharomyces cerevisiae* associates with the small nucleolar RNA snR5. Mol. Biol. Cell.

[37] Mayes A.E., Verdone L., Legrain P., Beggs J.D. (1999). Characterization of Sm-like proteins in yeast and their association with U6 snRNA. EMBO J..

[38] Kufel J., Allmang C., Verdone L., Beggs J.D., Tollervey D. (2002). Lsm proteins are required for normal processing of pre-tRNAs and their efficient association with La-homologous protein Lhp1p. Mol. Cell. Biol..

[39] Kufel J., Allmang C., Petfalski E., Beggs J., Tollervey D. (2003). Lsm Proteins are required for normal processing and stability of ribosomal RNAs. J. Biol. Chem..

[40] Pannone B.K., Kim S.D., Noe D.A., Wolin S.L. (2001). Multiple functional interactions between components of the Lsm2-Lsm8 complex, U6 snRNA, and the yeast La protein. Genetics.

[41] Pool M.R. (2005). Signal recognition particles in chloroplasts, bacteria, yeast and mammals (review). Mol. Membr. Biol..

[42] Saraogi I., Shan S.O. (2011). Molecular mechanism of co-translational protein targeting by the signal recognition particle. Traffic.

[43] Ciufo L.F., Brown J.D. (2000). Nuclear export of yeast signal recognition particle lacking Srp54p via the Xpo1p/Crm1p, NES-dependent pathway. Curr. Biol..

[44] Grosshans H., Deinert K., Hurt E., Simos G. (2001). Biogenesis of the signal recognition particle (SRP) involves import of SRP proteins into the nucleolus, assembly with the SRP-RNA, and Xpo1p- mediated export. J. Cell. Biol..

[45] Politz J.C., Yarovoi S., Kilroy S.M., Gowda K., Zwieb C., Pederson T. (2000). Signal recognition particle components in the nucleolus. Proc. Natl Acad. Sci. USA.

[46] Alavian C.N., Politz J.C., Lewandowski L.B., Powers C.M., Pederson T. (2004). Nuclear export of signal recognition particle RNA in mammalian cells. Biochem. Biophys. Res. Commun..

[47] Sinha K.M., Gu J., Chen Y., Reddy R. (1998). Adenylation of small RNAs in human cells. Development of a cell-free system for accurate adenylation on the 3'-end of human signal recognition particle RNA. J. Biol. Chem..

[48] Perumal K., Sinha K., Henning D., Reddy R. (2001). Purification, characterization and cloning of the cDNA of human SRP RNA 3' adenylating enzyme. J. Biol. Chem..

[49] Perumal K., Gu J., Reddy R. (2000). Evolutionary conservation of post-transcriptional 3' end adenylation of small RNAs: *S. cerevisiae* signal recognition particle RNA and U2 small nuclear RNA are post-transcriptionally adenylated. Mol. Cell. Biochem..

[50] Copela L.A., Fernandez C.F., Sherrer R.L., Wolin S.L. (2008). Competition between the Rex1 exonuclease and the La protein affects both Trf4p-mediated RNA quality control and pre-tRNA maturation. RNA.

[51] Schneider C., Kudla G., Wlotzka W., Tuck A., Tollervey D. (2012). Transcriptome-wide analysis of exosome targets. Mol. Cell.

[52] Leung E., Brown J.D. (2010). Biogenesis of the signal recognition particle. Biochem. Soc. Trans..

[53] Wlotzka W., Kudla G., Granneman S., Tollervey D. (2011). The nuclear RNA polymerase II surveillance system targets polymerase III transcripts. EMBO J..

[54] Tuck A.C., Tollervey D. (2013). A transcriptome-wide atlas of RNP composition reveals diverse classes of mRNAs and lncRNAs. Cell.

[55] Longtine M.S., McKenzie A., Demarini D.J., Shah N.G., Wach A., Brachat A., Phillippsen P., Pringle J.R. (1998). Additional modules for versatile and economical PCR-based gene deletion and modification in *Saccharomyces cerevisiae*. Yeast.

[56] Janke C., Magiera M.M., Rathfelder N., Taxis C., Reber S., Maekawa H., Moreno-Borchart A., Doenges G., Schwob E., Schiebel E. (2004). A versatile toolbox for PCR-based tagging of yeast genes: new fluorescent proteins, more markers and promoter substitution cassettes. Yeast.

[57] Van Nues R.W., Brown J.D. (2004). *Saccharomyces* SRP RNA secondary structures: a conserved S-domain and extended Alu-domain. RNA.

[58] van Nues R.W., Brown J.D. (2007). Distant segments of *Saccharomyces cerevisiae* scR1 RNA promote assembly and function of the signal recognition particle. J. Mol. Biol..

[59] van Nues R.W., Leung E., McDonald J.C., Dantuluru I., Brown J.D. (2008). Roles for Srp72p in assembly, nuclear export and function of the signal recognition particle. RNA Biol..

[60] Brown J.D., Hann B.C., Medzihradszky K.F., Niwa M., Burlingame A.L., Walter P. (1994). Subunits of the *Saccharomyces cerevisiae* signal recognition particle required for its functional expression. EMBO J..

[61] van Hoof A., Lennertz P., Parker R. (2000). Three conserved members of the RNase D family have unique and overlapping functions in the processing of 5S, 5.8S, U4, U5, RNase MRP and RNase P RNAs in yeast. EMBO J..

[62] Schneider C., Tollervey D. (2014). Looking into the barrel of the RNA exosome. Nat. Struct. Mol. Biol.

[63] Makino D.L., Baumgartner M., Conti E. (2013). Crystal structure of an RNA-bound 11-subunit eukaryotic exosome complex. Nature.

[64] Wasmuth E.V., Januszyk K., Lima C.D. (2014). Structure of an Rrp6-RNA exosome complex bound to poly(A) RNA. Nature.

[65] Wang Z., Castano I.B., De Las Penas A., Adams C., Christman M.F. (2000). Pol kappa: A DNA polymerase required for sister chromatid cohesion. Science.

[66] Liu J.J., Bratkowski M.A., Liu X., Niu C.Y., Ke A., Wang H.W. (2014). Visualization of distinct substrate-recruitment pathways in the yeast exosome by EM. Nat. Struct. Mol. Biol..

[67] Yoo C.J., Wolin S.L. (1994). La proteins from Drosophila melanogaster and *Saccharomyces cerevisiae*: a yeast homolog of the La autoantigen is dispensable for growth. Mol. Cell. Biol..

[68] Sinha K., Perumal K., Chen Y., Reddy R. (1999). Post-transcriptional adenylation of signal recognition particle RNA is carried out by an enzyme different from mRNA Poly(A) polymerase. J. Biol. Chem..

[69] Felici F., Cesareni G., Hughes J.M.X. (1989). The most abundant small cytoplasmic RNA of *Saccharomyces cerevisiae* has an important function required for normal cell growth. Mol. Cell. Biol..

[70] Granneman S., Kudla G., Petfalski E., Tollervey D. (2009). Identification of protein binding sites on U3 snoRNA and pre-rRNA by UV cross-linking and high-throughput analysis of cDNAs. Proc. Natl. Acad. Sci. USA.

[71] Granneman S., Petfalski E., Tollervey D. (2011). A cluster of ribosome synthesis factors regulate pre-rRNA folding and 5.8S rRNA maturation by the Rat1 exonuclease. EMBO J..

[72] Ozanick S.G., Wang X., Costanzo M., Brost R.L., Boone C., Anderson J.T. (2009). Rex1p deficiency leads to accumulation of precursor initiator tRNAMet and polyadenylation of substrate RNAs in *Saccharomyces cerevisiae*. Nucleic Acids Res..

[73] Kadaba S., Wang X., Anderson J.T. (2006). Nuclear RNA surveillance in *Saccharomyces cerevisiae*: Trf4p-dependent polyadenylation of nascent hypomethylated tRNA and an aberrant form of 5S rRNA. RNA.

[74] San Paolo S., Vanacova S., Schenk L., Scherrer T., Blank D., Keller W., Gerber A.P. (2009). Distinct roles of non-canonical poly(A) polymerases in RNA metabolism. PLoS Genet..

[75] Strub K., Fornallaz M., Bui N. (1999). The Alu domain homolog of the yeast signal recognition particle consists of an Srp14p homodimer and a yeast-specific RNA structure. RNA.

[76] Weichenrieder O., Stehlin C., Kapp U., Birse D.E., Timmins P.A., Strub K., Cusack S. (2001). Hierarchical assembly of the Alu domain of the mammalian signal recognition particle. RNA.

