# Supplementary material for: Integrity of SRP RNA is ensured by La and the nuclear RNA quality control machinery
Source: Nucleic Acids Res. 2014 Aug 26;42(16):10698–710. doi: 10.1093/nar/gku761 (PMC4176351; doi:10.1093/nar/gku761)
Supplement: SUPPLEMENTARY DATA [file supp_42_16_10698__index.html]

Integrity of SRP RNA is ensured by La and the nuclear RNA quality control machinery — Integrity of SRP RNA is ensured by La and the nuclear RNA quality control machinery — SUPPLEMENTARY DATA 

# Integrity of SRP RNA is ensured by La and the nuclear RNA quality control machinery

## SUPPLEMENTARY DATA

**Files in this Data Supplement:**

- SUPPLEMENTARY DATA
