# Supplementary material for: Integrity of SRP RNA is ensured by La and the nuclear RNA quality control machinery
Source: Nucleic Acids Res. 2014 Aug 26;42(16):10698–710. doi: 10.1093/nar/gku761 (PMC4176351; doi:10.1093/nar/gku761)
Supplement: SUPPLEMENTARY DATA [file supp_gku761_Leung_etal_supplementary.pdf]

SUPPLEMENTARY METHODS: Plasmid construction

Low (centromeric, pJNY347) and high (2μ, pJNY339) copy plasmids containing *LHP1* (with promoter and terminator regions, amplified from genomic DNA) were constructed in pRS315 (77) and pRS425 (78) respectively. *LHP1*^{F51A} was generated by PCR. 2 fragments were amplified from plasmid pJNY339 using T7 promoter primer plus BSAI51Aup (5'-CCAG*GGTCTC*GCGCAACGCCCTGTCATATGGAAAGTTG; BsaI site in italics altered codon 51 underlined) and T3 promoter primer plus BSAI51Ado (5'-CCAG*GGTCTC*TTGCGCACAACAGCG). These fragments were digested with BsaI, ligated and cloned into pRS425. Cloned fragments were sequenced to confirm accuracy.

SUPPLEMENTARY TABLE S1: Yeast strains used in this study.

Strain	Genotype	Reference/Source
JDY3	MATα, *trp1*, *ura3*, *ade2*, *his3*, *lys2*	(59)
JDY8	*sec65Δ::HIS3*, *[rho-]*, otherwise as JDY3	(79)
JDY44	*srp21Δ::HIS3*, *[rho-]*, otherwise as JDY3	(59)
JDY66	*srp14Δ::HIS3*, *[rho-]*, otherwise as JDY3	(59)
JDY97	*srp68Δ::HIS3*, *[rho-]*, otherwise as JDY3	(59)
JDY99	*srp72Δ::HIS3*, *[rho-]*, otherwise as JDY3	(59)
JDY103	*scr1Δ::HIS3*, *[rho-]*, otherwise as JDY3	(59)
JDY335	MATα, *srp14::HIS3*, *[rho-]*, *trp1-1*, *ura3-1*, *ade2-1*, *his3-11*, *-15, leu2-3, -112, can1-100*	This study
JDY483	MAT**a**, *trp1-1*, *ura3-1*, *ade2-1*, *his3-11*, *-15*, *leu2-3*, *-112*, *can1-100*, *scr1Δ::TRP1*, pRS316-*SCR1*	(57)
JDY596	*srp72::GFP-kanMX3*, otherwise as JDY3	This study
JDY618	MATα, *mtr4-1*, *ura3-52, lys2-801, pep4::HIS3, prb1-Δ1-6R, GAL+*	Alan Tartakoff
JDY721	MAT**a**, *ura3-1*, *ade2-1*, *his3-11*, *-15*, *leu2-3*, *-112*, *can1-100, trp1-1::*(*TRP1, GFP-MS2*)	This study
JDY726	MAT**a**, *ura3-1*, *ade2-1*, *his3-11*, *-15*, *leu2-3*, *-112*, *can1-100, trp1-1::*(*TRP1, GFP-MS2*), *scr1::MS2X6-HIS3*	This study
JDY895	MAT**a**, *his3Δ1*, *leu2Δ0*, *met15Δ0*, *ura3Δ0*	(80)
JDY898	*trf4Δ::kanMX6*, *HisMX6-pGAL-3HA::trf5*, otherwise as JDY895	(14)
JDY899	*air2Δ::kanMX4*, otherwise as JDY895	(11)

JDY900	*air1Δ::kanMX4*, otherwise as JDY895	(11)
JDY901	*air1Δ::kanMX4*, *air2Δ::natMX6*, otherwise as JDY895	(11)
JDY906	*rrp6Δ::HIS3MX6*, otherwise as JDY483	This study
JDY910	*lhp1Δ::HIS3MX6*, otherwise as JDY483	This study
JDY911	*trf4Δ::HIS3MX6*, otherwise as JDY483	This study
JDY912	*trf5Δ::HIS3MX6*, otherwise as JDY483	This study
JDY974	*rex1Δ::kanMX6* otherwise as JDY895	Euroscarf
JDY978	*lsm1Δ::kanMX4*, otherwise as JDY895	Jean Beggs
JDY981	*lsm6Δ:: hphMX6*, otherwise as JDY895	Jean Beggs
JDY982	*lsm7Δ::hphMX6*, otherwise as JDY895	Jean Beggs
JDY983	*lsm5Δ::hphMX6*, otherwise as JDY895	Jean Beggs
JDY984	*lsm4Δ::kanMX4*, otherwise as JDY895	Jean Beggs
JDY990	*natNT2-pGAL(S)::mtr4*, otherwise as JDY483	This study
JDY1015	*natNT2-pGAL(L)::rrp44*, otherwise as JDY483	This study
JDY1018	*natNT2-pGAL(L)::lhp1*, otherwise as JDY483	This study
JDY1028	*rex1Δ::natNT2*, otherwise as JDY483	This study
JDY1042	*lhp1Δ::HIS3MX6*, *rex1Δ::natMX6*, otherwise as JDY483	This study
JDY1058	*natNT2-pGAL(L)::lhp1*, *trf4Δ::HIS3MX6*, otherwise as JDY483	This study
JDY1083	*natNT2-pGAL(S)::mtr3*, otherwise as JDY483	This study
JDY1084	*natNT2-pGAL(L)::rrp42*, otherwise as JDY483	This study
JDY1093	*srp21::GFP-HIS3MX6*, otherwise as JDY895	This study
JDY1156	*air1Δ::kanMX6*, *air2Δ::natMX6*, *lhp1Δ::HIS3MX6*, otherwise as JDY895	This study
JDY1199	*lhp1Δ::natNT2* otherwise as JDY335	This study
JDY1201	*rrp6Δ::natNT2* otherwise as JDY335	This study
JDY1203	*trf4Δ::kanMX6* otherwise as JDY335	This study
JDY1239	*rex1Δ::kanMX4, srp14Δ::HIS3*, *[rho-]*, *ura3, his3, leu2 **	This study
JDY1241	*rrp44Δ::kanMX6*, pRS315-*rrp44-exo-HTP, srp14Δ:HIS3, ura3, his3, leu2 **	This study

CS-66	*rrp44Δ::kanMX6*, pRS315-*RRP44-HTP*, otherwise as JDY895	(3)
CS-77	*rrp44::kanMX6*, pRS315-*rrp44-exo-HTP*, otherwise as JDY895	(3)
CS-31	*trf4::HTP-URA3*, otherwise as JDY895	(50)

* The mating type of these strains has not been determined.

SUPPLEMENTAL TABLE S2 The 3' terminus of scR1.

The 3' terminus of scR1 was inferred by reverse transcription-PCR after ligating a 3'-blocked DNA oligonucleotide onto RNA isolated from the cells indicated. PCR products were sequenced directly and, where indicated, cloned and sequenced individually. The genomic sequence at the 3' end of *SCR1* is: …CATATTTTTTAACA… the run of 6 thymidines comprising the pol III terminator

Genotype	RT-PCR product	Cloned RT-PCR products	No. of clones
wild type	…CAUAUUUU(U)*	…CAUAUUUU	11
		…CAUAUUUUU	6
		…CAUAUUUUUU	1
		…CAUAUUU	1
		…CAUA	1
lhp1Δ	…CAUAU(U/A)(U/A)(U/A)	…CAUAAAAA	2
		…CAUAU	2
		…CAUAUAAA	1
		…CAUAUU	3
		…CAUAUUA	2
		…CAUAUUAA	1
		…CAUAUUAAA	4
		…CAUAUUU	4
		…CAUAUUUAA	4
		…CAUAUUUU	1
lhp1^{F51A}	…CAUAU(U/A)(U/A)(U/A)		
rex1Δ	…CAUAUUUUU(U)	…CAUAUUUU	1
		…CAUAUUUUU	8
		…CAUAUUUUUU	4
lhp1Δ, rex1Δ	…CAUAUU(U/A)(U/A)	…CAUAU	6
		…CAUAUAAA	2
		…CAUAUAAAA	1
		…CAUAUU	2
		…CAUAUUU	1
		…CAUAUUUAA	1
trf4Δ	…CAUAU(UUUU)	…AAUUGUGC$_{(420)}$[†]	1
		…CCAUCAGG$_{(462)}$	1
		…UUGGCGGU$_{(453)}$	3
		…CAUAU	2
		…CAUAUA	1
		…CAUAUU	2
		…CAUAUUU	5
		…CAUAUUUU	6
		…CAUAUUUUU	2
		…CAUAUUUUUA	1
		…CAUAUUUUUU	2
		…CAUAUUUUUUAACAUUUU[¶]	1
lhp1Δ, trf4Δ	…CAUAU(U)(U)(U)	…CUCGCACA$_{(475)}$	1
		…GUGUGUC$_{(507)}$	1
		…CAUAAAA	1
		…CAUAU	10
		…CAUAUU	1
		…CAUAUUU	2
		…CAUAUUUU	4

lhp1Δ, rrp6Δ	…CAUAU(U)(U)	…CAUAA<br>…CAUAAA<br>…CAUAAAA<br>…CAUAAAAA<br>…CAUAU<br>…CAUAUA<br>…CAUAUAA<br>…CAUAUAAA<br>…CAUAUUU	3<br>1<br>1<br>1<br>1<br>1<br>1<br>1<br>1
air1Δ, air2Δ	…CAUAU	…CAU<br>…CAUA<br>…CAUAU<br>…CAUAUAA<br>…CAUAUU<br>…CAUAUUU<br>…CAUAUUUUU	1<br>4<br>10<br>1<br>2<br>1<br>3
air1Δ	…CAUAUUUU(U)		
air2Δ	…CAUAUUUU(U)		
rrp6Δ	…CAUAU(U/A)(U/A)	…CAUAAAAA<br>…CAUAU<br>…CAUAUAAA<br>…CAUAUU<br>…CAUAUUAA<br>…CAUAUUU<br>…CAUAUUUU	1<br>3<br>2<br>2<br>2<br>1<br>1
rrp6^{D238N}	…CAUAU(U/A)(U/A)	…CAUAAAAAA<br>…CAUAU<br>…CAUAUAAA<br>…CAUAUUAAA<br>…CAUAUUUUU<br>…CAUAUUUUUU	1<br>4<br>2<br>1<br>1<br>1
rrp44-exo	…CAUAU(U)(U)	…CAU<br>…CAUA<br>…CAUAAAA<br>…CAUAU<br>…CAUAUU<br>…CAUAUUAA<br>…CAUAUUU<br>…CAUAUUUU<br>…CAUAUUUUAA<br>…CAUAUUUUU	1<br>1<br>3<br>10<br>2<br>1<br>4<br>2<br>1<br>5
rrp44-endo	…CAUAUUUU(U)		
rrp44-exo, rrp6Δ	…CAUAU(U)(U)	…CAUA<br>…CAUAU<br>…CAUAUU<br>…CAUAUUUU<br>…CAUAUUUUU	1<br>1<br>1<br>1<br>1
lsm4Δ	…CAUAUUUU(U)		
lsm5Δ	…CAUAUUUU(U)		
lsm6Δ	…CAUAUUUU(U)		
lsm7Δ	…CAUAUUUU(U)		

* Bracketed nucleotides indicate heterogeneity of the 3’ end with either alternative nucleotides at a given position, or that the sequence indicated the presence of a

particular nucleotide (such as the 5th U in wild type scR1) in only part of the cDNA product.

†numbers in brackets refer to final nucleotide of scR1 present in the clone sequenced.

¶ this clone extends beyond the terminator of scR1 into the next stretch of T's in the template.

Leung et al., Figure S1

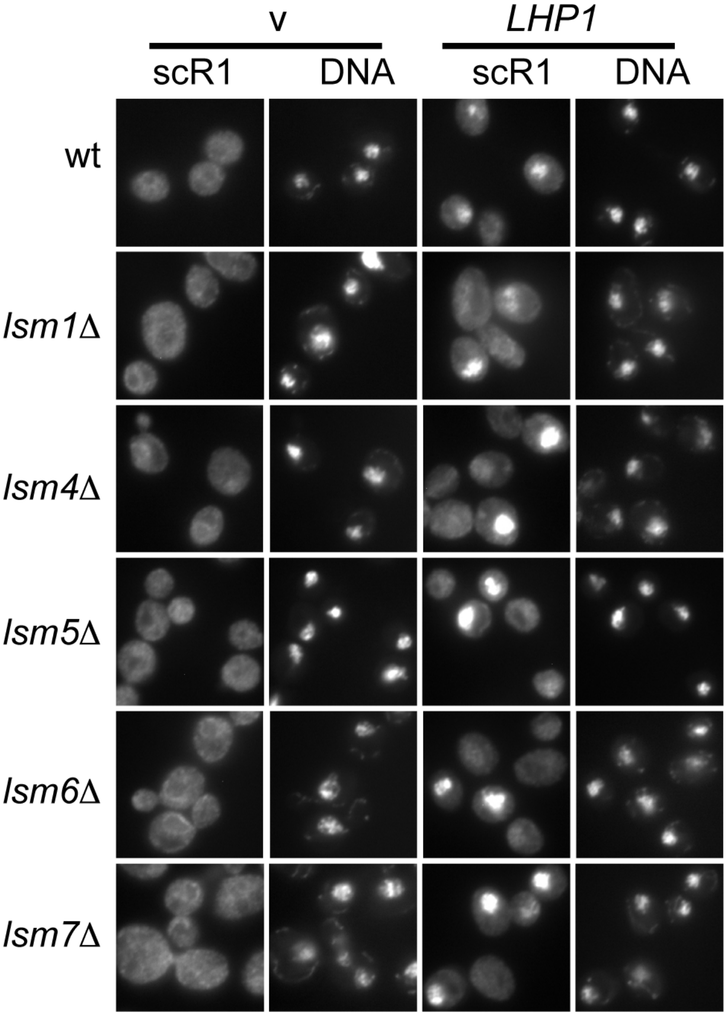

Figure S1 The nuclear LSM complex is not required for correct localisation of scR1, or for its nuclear accumulation on over-expression of Lhp1. Fluorescence *in situ* hybridisation (FISH) was carried out on the cells indicated, which contained high copy (2μ) plasmids that were either empty (v) or contained *LHP1*, using fluorescently labelled probe scR1A (scR1). DNA was stained with DAPI (DNA). Lsm1 is exclusively part of the cytoplasmic Lsm complex, and provided a control, whereas Lsm4-7 are components of both the cytoplasmic and

nuclear Lsm complexes. Individual channels are shown. Fluorescence images of cells were captured as described (Materials and Methods).

SUPPLEMENTARY REFERENCES

77. Sikorski, R.S. and Heiter, P.(1989) A system of shuttle vectors and yeast host strains designed for efficient manipulation of DNA in *Saccharomyces cerevisiae.* Genetics, **122**, 19-27.

78. Christianson, T.W., Sikorski, R.S., Dante, M., Shero, J.H. and Hieter, P. (1992) Multifunctional yeast high-copy-number shuttle vectors. Gene, **110**, 119-122.

79. Stirling, C.J. and Hewitt, E.W. (1992) The *Saccharomyces cerevisiae SEC65* gene encodes a component of the yeast signal recognition particle with homology to human SRP19. Nature, **356**, 534-537.

80. Brachmann, C.B., Davies, A., Cost, G.J., Caputo, E., Li, J., Hieter, P. and Boeke, J.D. (1998) Designer deletion strains derived from *Saccharomyces cerevisiae* S288C: a useful set of strains and plasmids for PCR-mediated gene disruption and other applications. Yeast, **14**, 115-32.
